# Fatal Asphyxia in the Head-down Position: An Uncommon Case of Accidental Work-related Death

**DOI:** 10.1097/PAF.0000000000001068

**Published:** 2025-09-03

**Authors:** Francesco D’Elia, Ugo Da Broi, Francesco Simonit, Alessio Cappelli, Sirio Cividino, Mauro Zaninelli, Rexson Tse, Jack Garland, Benjamin Ondruschka, Lorenzo Desinan

**Affiliations:** * Department of Medical Surgical and Health Sciences, School of Legal Medicine, University of Trieste, Trieste; † Department of Medicine, Legal Medicine, University of Udine, Udine; ‡ Department of Human Science and Quality of Life Promotion, S. Raffaele University, Rome, Italy; § Griffith University School of Medicine and Dentistry, Southport; ∥ Forensic Pathology and Coronial Services, Brisbane, Queensland, Australia; ¶ Institute of Legal Medicine, University Medical Center Hamburg-Eppendorf, Hamburg, Germany

**Keywords:** asphyxial deaths, positional asphyxia, death in head-down position, agricultural fatalities, forensic pathology

## Abstract

Positional asphyxia is a rare but potentially fatal condition where an individual’s body assumes a position interfering with normal respiratory movements and leading to asphyxiation. We report a case of an 83-year-old farmer trapped in the tank of a vineyard spray atomizer while attempting maintenance and stuck in the opening of the tank with his upper body inside and legs outside. He was unable to extricate himself, and rescuers, who arrived 2 hours later, found the victim dead. The forensic autopsy showed patterned external injuries corresponding to the edge of the opening of the tank, internal signs of asphyxia such as pulmonary congestion and pleural petechiae, in the absence of major traumatic injuries. The cause of death was confirmed as positional asphyxia due to the head-down position and abdominal compression, which impaired breathing. The victim disregarded safety protocols, which prohibit entry into the loading tank of the sprayer, which is classified as a confined space by manufacturers. This incident highlights the importance of adhering to safety guidelines in all working routines, using appropriate personal protective equipment (PPE) and following proper maintenance procedures and adequate safety practices. Enhanced safety measures and safer equipment design are crucial to prevent similar occurrences.

Death due to positional asphyxia is an uncommon accidental event occurring when the victim’s body assumes a position which can compromise the effective respiratory dynamics, and does not occur when the body is directly compressed by external forces causing the “crush asphyxia.”^1^ Fatal positional asphyxia may occur in different contexts, such as cases involving occupational accidents or home accidents, especially in the presence of drunkenness, drug intoxication, or disabilities,^2^ and is a very rare event among agricultural accidents. Medicolegal diagnosis of fatal positional asphyxia may be difficult and should include investigating the factual circumstances and the original positioning of all the body segments, with the exclusion of other possible significant underlying causes of death.^3^ We present a fatal agricultural accident due to positional asphyxia during the maintenance of a vineyard spray atomizer machine and discuss the diagnostic approach to confirm the death was accidental and to exclude the possibility of homicide or suicide.

## CASE REPORT

**FIGURE 1:**
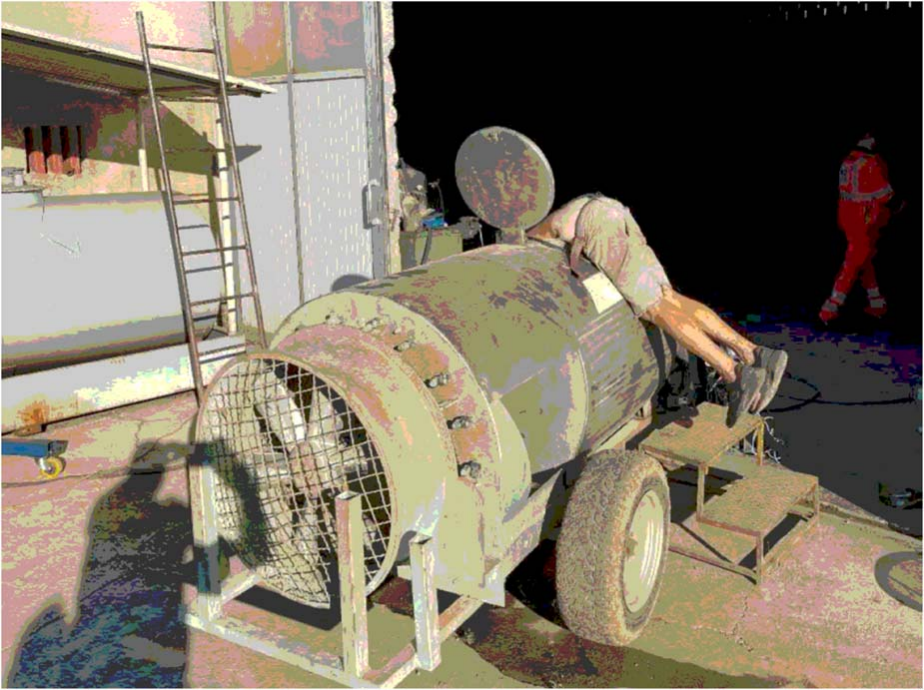
Original position of the body as it was found by the rescuers.

**FIGURE 2:**
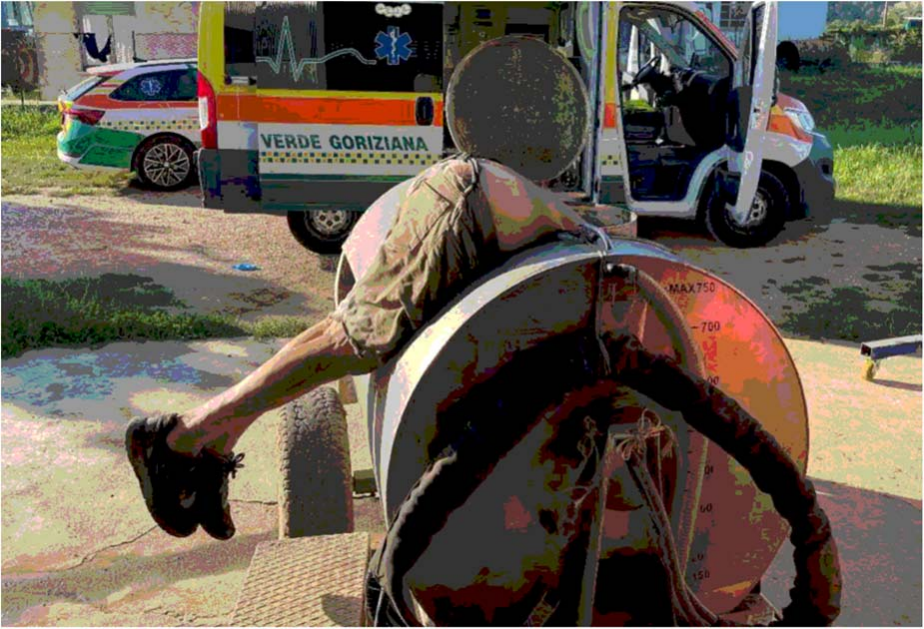
Original position of the body as it was found by the rescuers.

**FIGURE 3:**
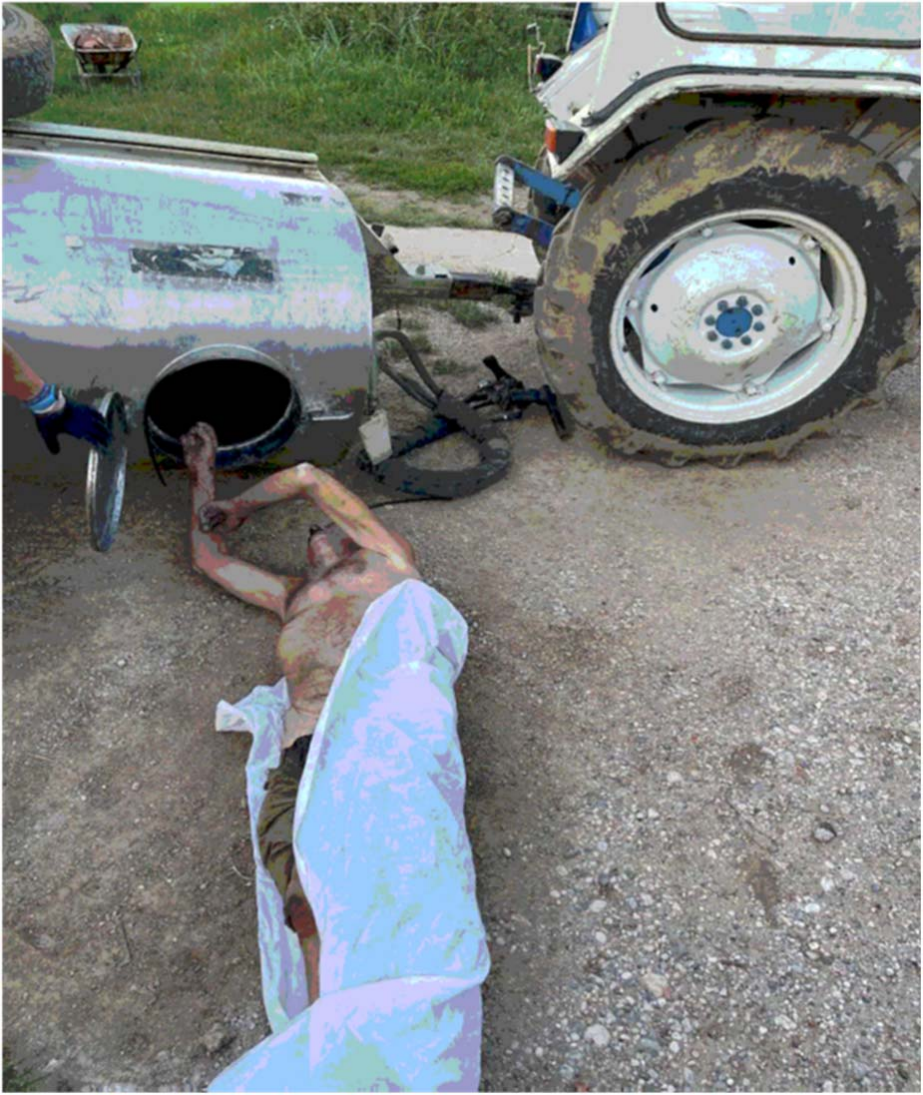
Extraction of the victim’s body after rotating the tank with a crane.

An 83-year-old man with no pre-existing medical conditions was working with his brother at their farm. He entered the upper and large opening (40 cm in diameter) of a high-volume tank of a vineyard spray atomizer to make some repairs, but became wedged at the waist, with the upper body pending inside the tank and his legs outside, so he asked his brother who was there for help. The brother, affected by serious intellectual disability since birth, as later certified by a forensic psychiatric assessment, did not realize the emergency of the situation and called for medical rescue more than 2 hours after the victim had gotten stuck (this time interval was verified by the Police when investigating circumstantial data). Unfortunately, no other witnesses were present at the scene when the event occurred. When medical rescuers arrived, the man was found dead with the upper part of his body (head and upper limbs) inside the tank, while his legs were hanging down outside (Figs. 1, 2). The body was stuck with the edge of the opening circumference pressing on his abdomen, so that rescuers were unable to extract it. Upon arrival, before extracting the body, the firefighters immediately tested the air in the tank with a gas detector, finding that there was no carbon monoxide and the oxygen concentration was normal (20.8%). The tank was empty because the cap on the bottom was open, and a hammer was found inside, consistent with the fact that the subject was making repairs. Pictures of the unaltered scene were taken by rescuers before the medicolegal inspection of the death scene. In order to remove the body stuck in that position without damaging it, it was necessary to lift and rotate the tank with a crane (Fig. 3).

**FIGURE 4:**
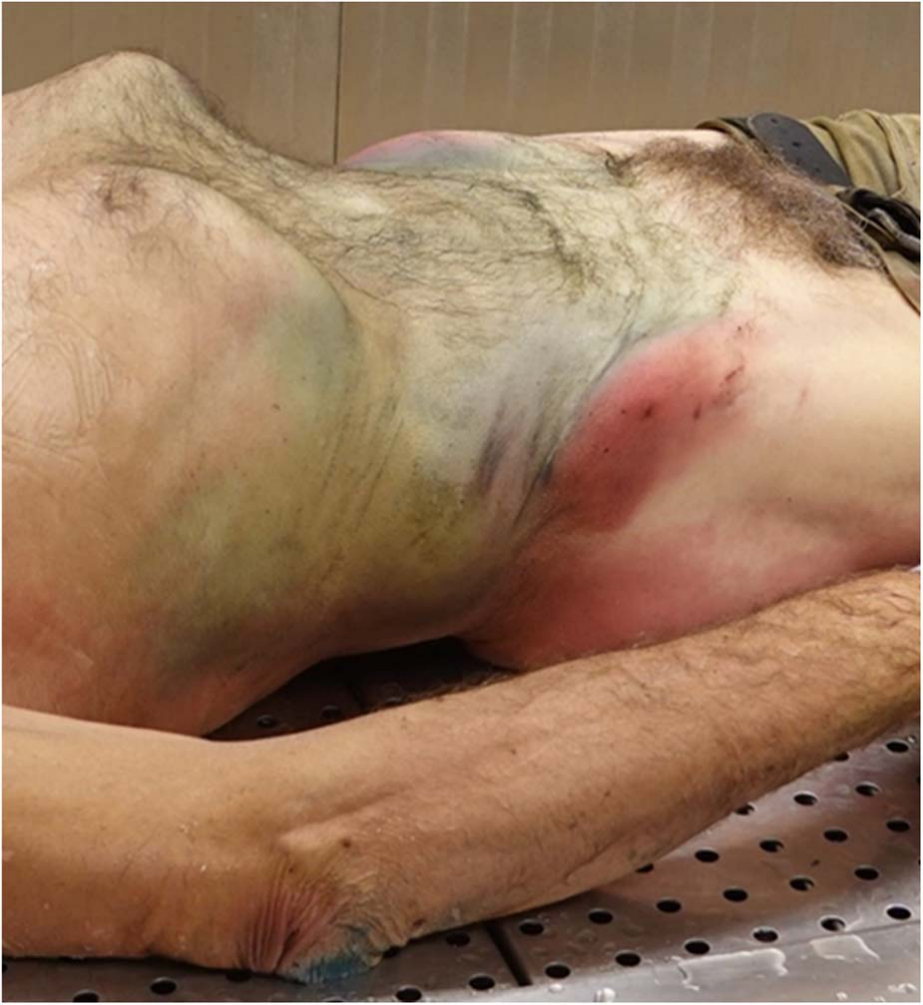
Patterned bruises and abrasions at the autopsy examination.

**FIGURE 5:**
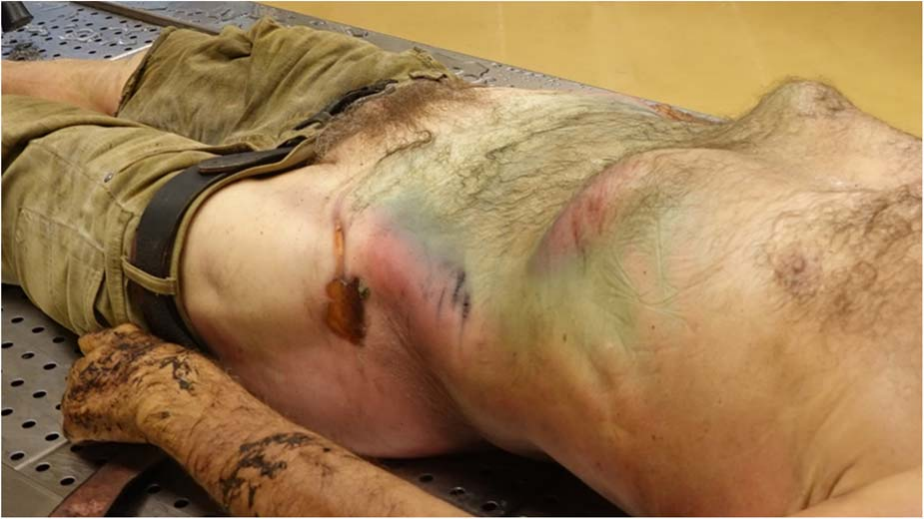
Patterned bruises and abrasions at the autopsy examination.

The main autopsy findings were the following:

- Fixed hypostasis in the head, upper chest, shoulders and arms;
- Clotted blood oozing out of the nose;
- On the abdominal sides, blurred bruises and linear abrasions, transverse and parallel to each other;
- Below the left iliac crest, a rectangular abrasion, continuing transversely in the hypogastric region with an excoriated streak <1 cm wide (Figs. 4, 5), unrelated to any internal traumatic injury found at autopsy;
- Congested brain superficial vessels and venous engorgement, mild cerebral edema, and disseminated petechiae in the white matter, including the brainstem;
- Blackish fluid material in the esophagus;
- Increased lung volume and weight (right 880 g, left 800 g; normal: right 360 to 570 g, left 325 to 480 g)^4^ with petechiae in the pleural fissures, severe congestion and edema;
- Airways free from any obstructions;
- Hypertrophic heart without other abnormalities and normal coronary arteries.

Histological examinations showed congestion of small vessels in the white matter of the brain, pulmonary edema and congestion, mild focal interstitial myocardial fibrosis, and chronic interstitial nephritis.

Toxicological analyses were negative for alcohol, psychoactive and hazardous substances, including pesticides or fertilizers. No chemicals or hazardous substances were found in the tank by the Firefighters Chemical Laboratory Unit, and the internal surface of the tank was dry and free from residues of any material or chemical compounds. Usually, the deceased used the spray atomizer exclusively for applying copper-based fungicide (copper sulfate) in the vineyard, but at the moment of the fatality, he was merely conducting maintenance operations inside the tank using a hammer.

After autopsy, the cause of death was certified as asphyxia in the head-down position, with the pressure on the upper abdomen impeding breathing movements of the diaphragm. Other relevant causes of death were excluded, such as stroke, myocardial infarction, pulmonary embolism, and major internal bleeding due to the absence of specific autopsy and histological-related evidences.

The manner of death was a fatal accident at work, in the absence of traumatic lesions attributable to third parties or toxicological causes.

### Technical Specifications, Functioning, and Operating Procedures of Spray Atomizers

The machine involved in this fatality was a vineyard spray atomizer. There were no identifying elements (labels, numeric codes) indicating a specific manufacturer, but based on the geometries and characteristics of the sprayer, its production date was estimated to be around the 1990s. The primary function of the machine is to spray pesticides in vineyards. The spray atomizer is coupled with a tractor (a towed machine) that provides mechanical and hydraulic power.

The spray atomizer mainly consists of 4 components:

- Machine frame
- Mixture distribution circuit ending with nozzles
- Distribution pump
- Fan

**FIGURE 6:**
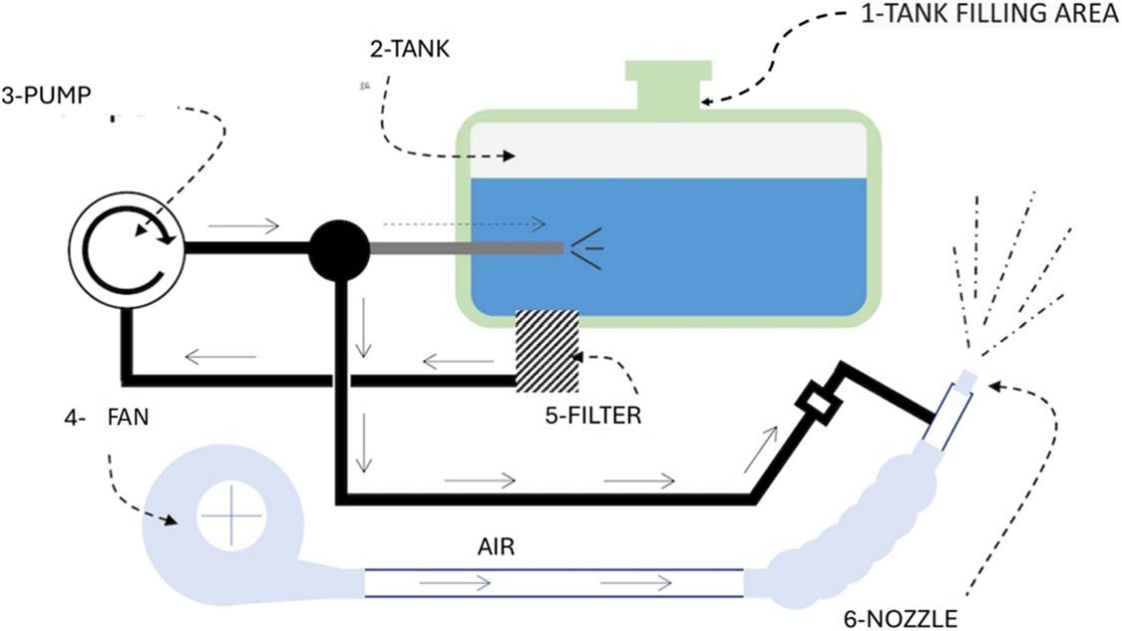
Machine operation diagram and loading area.

Figure 6 shows the schematic diagram of the sprayer’s operation.

The operator manually loads the machine through the tank filler neck (position 1, as shown in Fig. 6) with water mixed with the active ingredient. In more recent machines, the active ingredient and water are loaded separately and mixed by a mixing unit, integrated in the machine. Manufacturers of machines classify the loading tank of the sprayer as a confined space and, according to the standards, allow only maintenance technicians to go inside it.

According to the literature, there are primarily five risk factors that could lead to fatal incidents in the use of vineyard sprayers:^5–10^

- Risks generated by mechanical factors, such as the uncovered transmission of the tractor’s power take-off shaft.^5,6,11^
- Risks associated with the improper use of pesticides.^12,13^
- Risks due to road circulation.^5,6,14^
- Risks of vehicle overturning, especially in hilly areas or regions with slopes.^5,6,14^
- Risks due to confined spaces.^15,16^

A tank should be inspected (extraordinary maintenance operation) exclusively if:

Qualified personnel carried out this activity;
There are handles in the tank that allow the operator to get out;
The operator wears harnesses to avoid falling inside the tank and is equipped with suitable PPE;
The tank is free from pesticide/fertilizer residues;
The operator is accompanied by another skilled worker.^5–10^

All the above-reported requirements have not been followed in the case under study, leading to an avoidable fatal outcome.

## DISCUSSION

Positional asphyxia is different from other forms of asphyxia. In cases of asphyxia due to enclosed and confined spaces the fraction of available oxygen in ambient air is reduced, while in cases of crushing asphyxia (so called Perthes asphyxia), the agent mechanism is an external force compression of the chest or abdomen by heavy objects.^17,18^

Positional or postural asphyxia occurs when the body assumes, for a prolonged period of time, a position that ends up interfering with adequate respiratory movements, leading to impaired respiration and lung hypoventilation.^1–3^

Sometimes the above 3 different asphyxial mechanisms may coexist in the same event.

There are several positions the body can assume that can lead to death from positional asphyxia, such as prone immobilization (restrained prone), head-down, “jack-knife” position, reverse suspension, wedging of the body in restricted spaces, hyperflexion or hyperextension of the neck with partial or complete obstruction of the external airways.^2,19–22^

Most authors use the following 6 definition criteria proposed by Byard et al^2^ for positional asphyxia:

1. Victim in a position that does not allow for adequate respiration (including restrictive or confining positions, flexion of the head onto the chest, external airway obstruction and neck compression);
2. Victim inadvertently placed him or herself in the situation;
3. Victim unable to extricate him or herself from the situation (including conditions of intoxications or dementia);
4. No evidence of internal airway obstruction;
5. No evidence of inhalation of suffocating gases;
6. In the presence of underlying organic diseases, they must be unrelated to the terminal episode or, alternatively, may have predisposed to positional asphyxia.

Findings which may be observed during autopsy investigations for death by positional asphyxia may sometimes include, for example, congestion of the face and/or neck, facial and conjunctival petechiae, pulmonary congestion and edema.^3,23^

However, this case presents features of a peculiar type of positional asphyxia: a death in a head-down position. This is a rare event, which, as observed during the inspection of the death scene, was confirmed by the evidence of the body in an inverted position with the upper part of the body hanging downward. It is considered a distinct entity from positional asphyxia, with higher occurrence in the elderly population, with strong male predominance and alcohol intoxication as a frequent contributing factor, especially in younger victims.^24,25^ In fact, acute alcohol and/or drug intoxications are a crucial predisposing factor for deaths due to positional asphyxia, for the obvious reason that heavily intoxicated subjects might collapse or fall into confined situations or postures, where they are unable to breathe properly.^26–28^

In most cases of positional asphyxia, the manner of death has been reported as accidental because the victim results to be trapped, drunk or drugged and therefore unable to escape from life-threatening situations.^1,2,29^

Pathophysiology of death in the head-down position is not supposed to be simply related to asphyxia, because of the important role of heart failure in this particular mode of death. The inverted position often causes an excessive burden of work for the heart, which shows an abnormal increase in the presystolic volume load of cardiac chambers, causing rapid congestive heart failure. This is usually in association with increased cerebral venous congestive stasis and increased cerebral vascular resistances opposing arterial flow, with consequent hypoxic early loss of consciousness. Moreover, the abdominal organs in the head-down position may exert increased pressure on the diaphragm based on gravity, contributing to its pathological blocking and impaired respiration.^25^

In the case we report, the subject was elderly (83 years old) and the heart was found to be hypertrophic; this may also have contributed to making the subject vulnerable to death from the head-down position, underlying the role of heart failure in this mode of death. Therefore, as the individual was entrapped for a duration sufficient to cause respiratory insufficiency, this feature also satisfies the sixth definition criterion of positional asphyxia proposed by Byard et al.^2^

It is really difficult to distinguish the exact type of asphyxia based only on autopsy findings, so it is mandatory to support investigations with circumstantial data, death scene inspection data and by excluding other causes of death.

Some indicative elements or lesions found on the body may suggest that something impeded respiratory movements.^3,30^ As described in our case, there were patterned injuries such as “tramline” bruises and abrasions on the abdominal sides, that clearly corresponded to the edge of the opening of the tank in which he was found stuck. This observation was essential because there were no other significant pathological elements, such as major traumatic injuries. The mechanisms that had contributed to cause the death of the subject from positional asphyxia were the head-down position and the abdominal compression maintained over a long period of time, leading to impaired and finally blocked respiratory movements.

On the basis of the death scene inspection and photographic reconstruction, it is plausible to assume that the cause of death could be attributed to the improper action of the operator. Probably, he intended to inspect the tank from inside, an operation that is not permitted. The reasons for an inspection could be various; however, the most plausible could be the removal of objects or deposits that may have impeded the correct functionalities of the machine, like the hammer, which was later found inside the tank.

As highlighted by the photographic reconstruction, the operator used a ladder to reach the access point to the tank. After he had inserted the upper part of his body into the tank, he was unable to get out because there was nothing inside to grab onto to push himself out, while his arms were dangling into the tank. Moreover, he was stuck, so that neither rescuers nor firefighters were able to extract him; a crane was necessary to turn the tank on the side and extract the body without damaging it.

Work-related deaths by asphyxia have previously been described in the literature, and the main causes include asphyxiation from toxic or oxygen-deficient atmospheres, confined spaces, and crushing asphyxia.^31^ Serious agricultural accidents and fatalities occur in a variety of working contexts involving specific activities, but asphyxiation is not a common cause of accidental deaths in this scenario. It is usually due to “crush asphyxia” in tractor operators, which may be crushed when the vehicle rolls on uneven terrain, or it is also due to entrapment and asphyxiation in grain storage structures during unloading of the structures.^32,33^ In general, work-related fatalities from positional asphyxia in farming are unusual, especially in the Italian but also worldwide context.^34,35^

The victim’s occupation is of particular interest, because farmers are usually self-employed workers who are therefore exempt from several precautionary measures taken by organizations and government agencies to prevent accidents, such as the mandatory worker protection standards, compliance with specific training requirements and accident reporting procedures. In particular, it is the deficiency in safety training and the common working-alone scenario making it difficult to report and consequently reduce the number of farm accidents and fatalities.

### Concluding Remarks

The article highlights that the analyzed incident is representative of fatal accidents in the agricultural sector, where operators frequently disregard established safety procedures. The operator’s decision to inspect the interior of the sprayer’s tank, a procedure prohibited by manufacturers, led to a fatal outcome due to asphyxiation from postural asphyxia in a head-down position. The absence of proper personal protective equipment (PPE), such as harnesses, breathing apparatuses, and the failure to adhere to critical safety measures, such as having a skilled second operator present, further contributed to the incident.

This unique case is noteworthy because reports of fatal consequences in the agricultural sector due to positional asphyxia inside the tank of a spray atomizer, resulting from violations of occupational safety regulations well described in the literature.^15,16^

Moreover, the case reported underscores the importance of:

- Planning a detailed analysis of the death scene, related circumstances, and the technical characteristics of machinery involved, as well as autopsy and toxicological examination in case of investigations involving agricultural fatalities due to incorrect use of spray atomizers;
- Compelling for stricter adherence to safety guidelines in order to prevent accidents often attributable to human errors and/or significant underestimation of the associated risks.

Therefore, the reported fatality reinforces the need for rigorous operative directives and innovative safety devices in order to avoid incongruous and dangerous operations inside the tank of spray atomizers.
